# A New Zearalenone Biodegradation Strategy Using Non-Pathogenic *Rhodococcus pyridinivorans* K408 Strain

**DOI:** 10.1371/journal.pone.0043608

**Published:** 2012-09-25

**Authors:** Rókus Kriszt, Csilla Krifaton, Sándor Szoboszlay, Mátyás Cserháti, Balázs Kriszt, József Kukolya, Árpád Czéh, Szilvia Fehér-Tóth, Lívia Török, Zsuzsanna Szőke, Krisztina J. Kovács, Teréz Barna, Szilamér Ferenczi

**Affiliations:** 1 Laboratory of Molecular Neuroendocrinology, Institute of Experimental Medicine, Budapest, Hungary; 2 Department of Environmental Protection and Safety, Szent István University, Hungary; 3 Soft Flow Hungary R&D Ltd. Pécs, Hungary; 4 University of Pecs, Faculty of Medicine, Department of Biophysics, Pécs, Hungary; 5 Department of Genetics and Applied Microbiology, University of Debrecen, Hungary; Clermont Université, France

## Abstract

Zearalenone (hereafter referred to as ZEA) is a nonsteroidal estrogenic mycotoxin produced by several *Fusarium spp*. on cereal grains. ZEA is one of the most hazardous natural endocrine disrupting chemicals (EDC) which induces hyper estrogenic responses in mammals. This can result in reproductive disorders in farm animals as well as in humans. Consequently, detoxification strategies for contaminated crops are crucial for food safety. In this study we have developed a bacterial based detoxification system using a non-pathogen *Rhodococcus pyridinivorans* K408 strain. Following 5 days treatment of ZEA with *R. pyridinivorans* K408 strain HPLC analyses showed an 87.21% ZEA-degradation efficiency of the bacterial enzyme systems. In another approach, the strain biotransformation ability has also been confirmed by a bioluminescent version of the yeast estrogen screening system (BLYES), which detected an 81.75% of biodegradability of ZEA, in a good agreement with the chemical analyses. Furthermore, the capacity of *R. pyridinivorans* to eliminate the estrogenic effects of ZEA was tested by using an immature uterotrophic assay. Prepubertal female rats were treated with vehicle (olive oil), 17β-estradiol, ZEA (0.1-1-5-10 mg/kg body weight) and LB broth containing 500 mg/l ZEA that has already been incubated with or without *Rhodococcus pyridinivorans* K408 strain. Uterine weights were measured and the mRNA level changes relating to apelin, aquaporin 5, complement component 2, and calbindin-3 genes were measured by qRT-PCR. These genes represent the major pathways that are affected by estromimetic compounds. Zearalenone feeding significantly increased the uterus weight in a dose dependent manner and at the same time upregulated complement component 2 and calbindin-3 expression as well as decreased apelin and aquaporin 5 mRNA levels comparable to that seen in 17β-estradiol exposed rats. In contrast, LB broth in which ZEA was incubated with *Rhodococcus pyridinivorans* K408 *prior to* the feeding did not display any estrogenic effect neither on uterine weight nor on the expression of estrogen-regulated genes. Consequently, the identification of *Rhodococcus pyridinivorans* K408 strain in ZEA biodegradation proved to be a very efficient biological tool that is able to eliminate the complete estrogenic effects of ZEA. It is also remarkable that this biotransformation pathway of ZEA did not result in any residual estrogenic effects.

## Introduction

Zearalenone (ZEA, previously known as F-2 toxin) is an estrogenic mycotoxin produced by several *Fusarium spp*. species.(*F. graminearum (Gibberella zeae), F. culmorum, F. equiseti, F. crookwellense,* and *F. semitectum*) [1]. ZEA is found worldwide in a number of cereal crops such as maize, barley, oats, rice and sorghum and it is heat stable, hence the processing of raw materials in food industry does not eliminate ZEA and the toxin remains in the end-product, in bread for example [2], [3]. Consequently ZEA represents both a health risk and an economic problem. The rapid growth of the human population requires the development of a cost effective and safe decontamination processes for raw materials and feed to reduce the post harvesting loss.

ZEA binds to both forms of estrogen receptor, ER-α and ER-β (ERs) in vitro with similar affinity [4]–[5]. In vivo, ZEA is rapidly absorbed after oral administration [2] and metabolized into two major metabolites, α-zearalenol (α-ZOL) and β-zearalenol (β-ZOL), which are also ligands of ER [6]. The metabolite α-ZOL shows higher estrogenicity than ZEA, by contrast β-ZOL was less effective in MCF7 proliferation test [7].

Acute effects of ZEA are vulva vaginitis and enlarged reproductive tracts in females and atrophy of the seminal vesicles and testes in males [8]. The female reproductive system is more sensitive to ZEA than males, mainly in the prepubertal age. Prepubertal ZEA administration causes accelerated vaginal opening, persistent estrus and sterility [9]. Furthermore, exposure of zearalenone has the effect of reducing fertility and litter size in domestic animals [10] and ZEA mycoestrogens have been suspected as triggering factors for central precocious puberty in girls [11]. It should be noted that ZEA has a relatively low acute toxicity (oral LD 50 values of >2000–20000 mg/kg body weight.) after oral administration in mice, rats and guinea pigs. Pigs and sheep appear to be more sensitive than rodents; in controlled studies with a well-defined exposure to multiple ZEA doses [2]. ZEA and derivatives showed similar estrogenic properties, with the exception of α-ZOL which induced a higher estrogenic activity [12].

**Figure 1:**
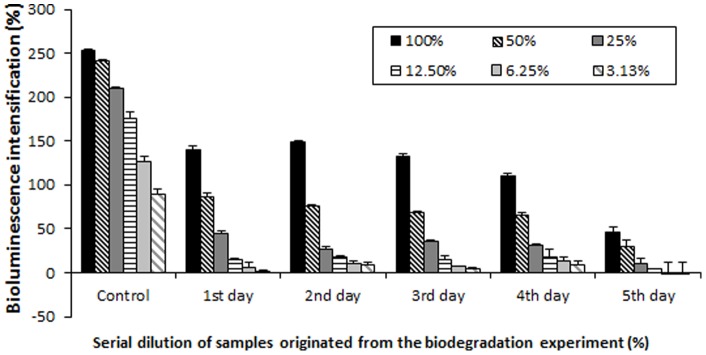
Remained estrogen effect in the zearalenone metabolism of *Rhodococcus pyridinivorans* analysed by the bioluminescens yeast reporter system (BLYES). The results show that the incubation with *Rhodococcus pyridinivorans* K408 decreased the estrogenic activity of zearalenone. After 5 days, remained estrogen effect was 81.75% compared to control. The results of the BLYES based estrogenic biotest showed good correlation with ZEA concentration measurement via HPLC-FLD.

To eliminate health- and economic threats that occur as a result of these toxins, detoxification procedures are needed as a matter of urgency. There are many microbes that are capable of degrading mycotoxins [13], for instance, *actinomycete* strains – especially *rhodococci* [14], are the best aflatoxin degraders. ZEA has also been shown to be eliminated by *Acinetobacter sp.* SM04 [15] and also by *Pseudomonas putida* ZEA-1 [16]. Recently, several microbes from hydrocarbon contaminated sites have been isolated in Hungary and deposited in the strain collection of Agruniver Holding Ltd. (*Rhodococcus pyridinivorans* K408 strain: GenBank accession number: jx021450). The mycotoxin degradation capacity of these strains has been confirmed in previous studies [17]. As such, the possible endocrine effects of the degraded ZEA products require analysis by effective testing methods.

**Figure 2:**
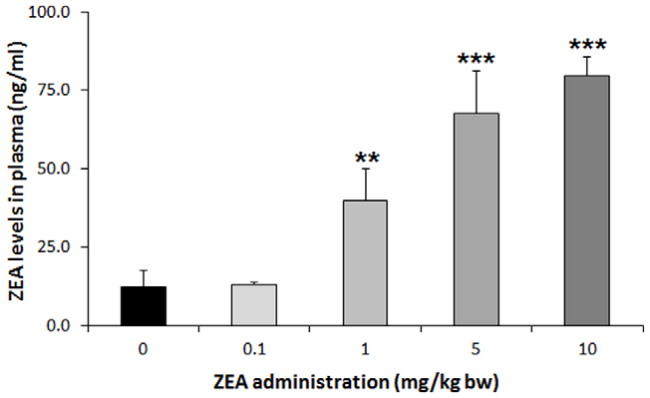
Blood plasma ZEA concentrations in *Experiment 1*. Blood plasma ZEA concentrations in female immature rats after 3-day exposure to different dosage of ZEA (0.1–10 mg/kg bw) was measured by competitive fluorescent-microsphere based immunoassay (CFIA). The results show that there was a dose dependent increase in plasma ZEA concentration following oral administration. Blood plasma levels showed a strong positive correlation (R  = 0.919186) with uterus weight in a dose dependent manner. (**) Significantly different from control (p<0.001) (n = 6–7). (***) Significantly different from control (p<0.001) (n = 6–7).

For the measurement of estrogenic effects, the estrogen bioindicator yeast strain YZRM7 [18] or HitHunter EFC estrogen chemiluminescence assay kit (DiscoveRx/Amersham Biosciences) could be utilised. Recently, a fast, simple, cost-effective luminescence based yeast estrogen screen (BLYES) was developed to reveal harmful biological effects of EDCs [19], [20]. By integrating a human estrogen receptor into the genome, along with bacterial *lux* cassette on plasmids, the *Saccharomices cerevisiae* BLYES strain has been engineered. The presence of moieties that bind to the estrogen receptor result in an increased bioluminescence of the tester strain, which seems to be a valuable tool for analyzing ZEA levels. In our study the BLYES test was performed to detect the estrogen effects of the metabolites produced.

**Figure 3:**
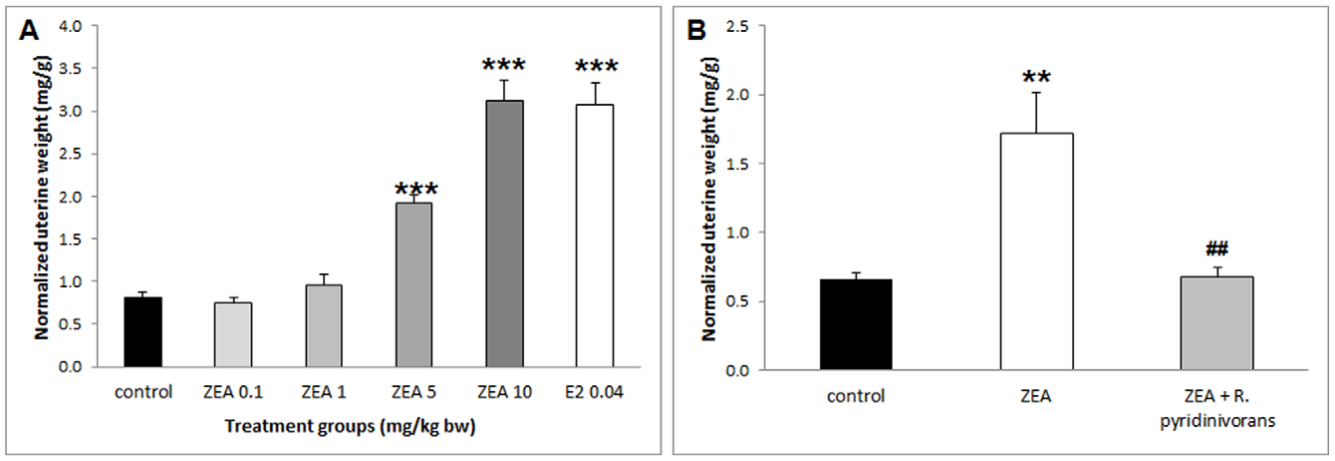
Uterine weights of *Experiment 1* and *Experiment 2.* Uterotrophic response expressed as relative uterus weight (uterus weight/body weight) in female immature rats after 3-day exposure to E2 (0.04 mg/kg bw) and ZEA (0.1–10 mg/kg bw) (**A,** *Experiment 1*) and after 3-day exposure to ZEA-contaminated LB media with and without incubation with *R. pyridinivorans* K408 (**B,** *Experiment 2*). (***) Significantly different from control in *Experiment 1* (p<0.001) (n = 6–7). (**) Significantly different from control in *Experiment 2* (p<0.01). (##) Significantly different from ZEA in *Experiment 2* (p<0.01) (n = 6–8).

However, apart from the *in vitro* screening methods, *in vivo* studies are also needed to investigate biodegradation efficiency directly on the neuroendocrine system. For this purpose the application of a rodent based uterotrophic bioassay can be the most suitable approach, in which two markers are measured: the uterine weight and changes in the estrogen-dependent gene expression in the uterine tissue. Uterine weight gain after treatment with different ECDs has been previously described [21] It has been shown that estrogen and compounds with estrogen-like effects regulate several pathways in uterine tissue. The main regulated pathways are the remodeling of extracellular matrix, alternative complement activation, cell proliferation and estrogen-mediated calcium signaling. As such, these estrogen-receptor-mediated alterations in gene expression, demonstrated by a microarray study, and the results, which can be validated by quantitative real-time PCR measurement [22] are useful markers to elucidate the biodegradation processes.

**Figure 4:**
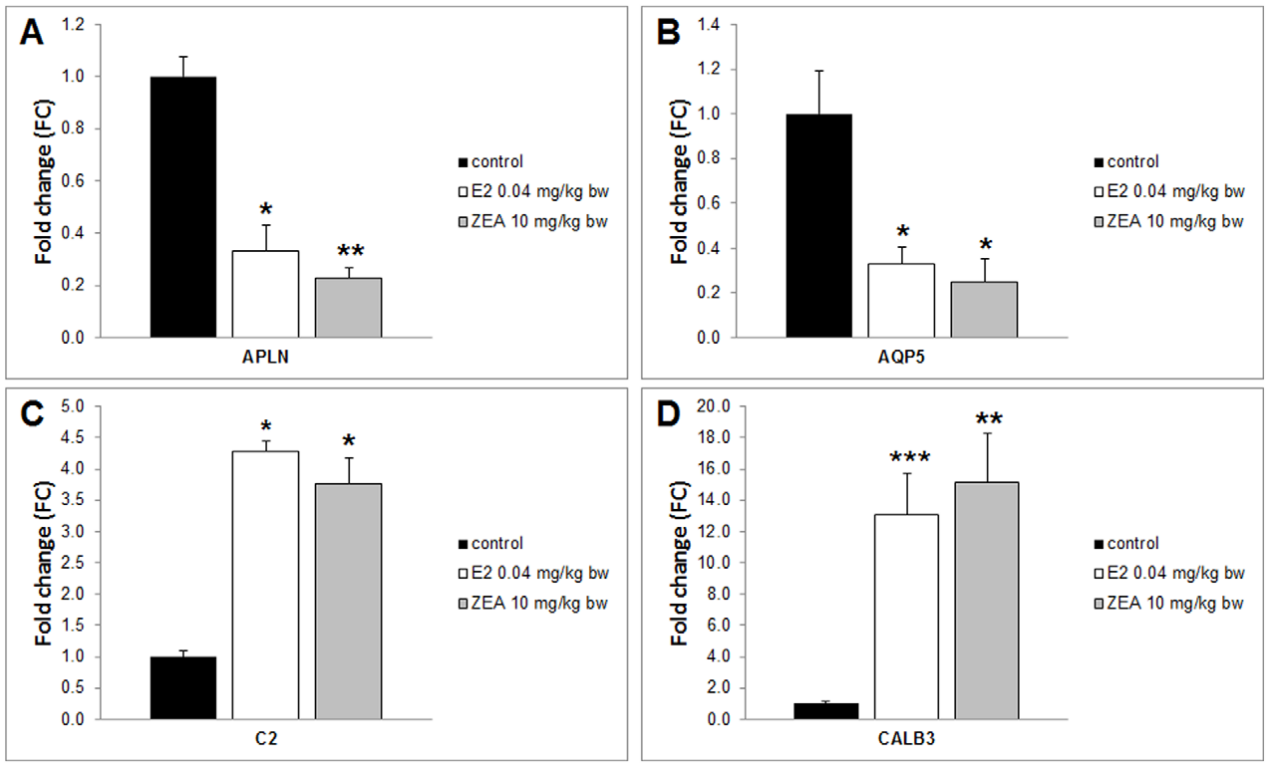
Gene expression of apelin, aquaporin 5, complement component 2 and calbindin 3 in *Experiment 1*. Gene expression of apelin (APLN) and aquaporin 5 (AQP5) decreased and complement component 2 (C2) and calbindin 3 (CALB3) increased significantly both by E2 0.04 mg/kg bw and ZEA 10 mg/kg bw 3-day-long treatment. (*) Significantly different from control (p<0.05). (**) Significantly different from control (p<0.01) (n = 6–7). (***) Significantly different from control (p<0.001) (n = 6–7).

In this study our aim was to develop an efficient biotransformation procedure for ZEA degradation with *Rhodococcus pyridinivorans* K408 strain. At the same time we targeted the development of a comprehensive monitoring *in vitro* -and *in vivo* system, in which both the level of the degraded ZEA and the remaining effect of the non-degraded ZEA on the neuroendocrine system can be evaluated. The *in vivo* measurements comprised an uterotrophic bioassay with prepubertal female rats.

**Figure 5:**
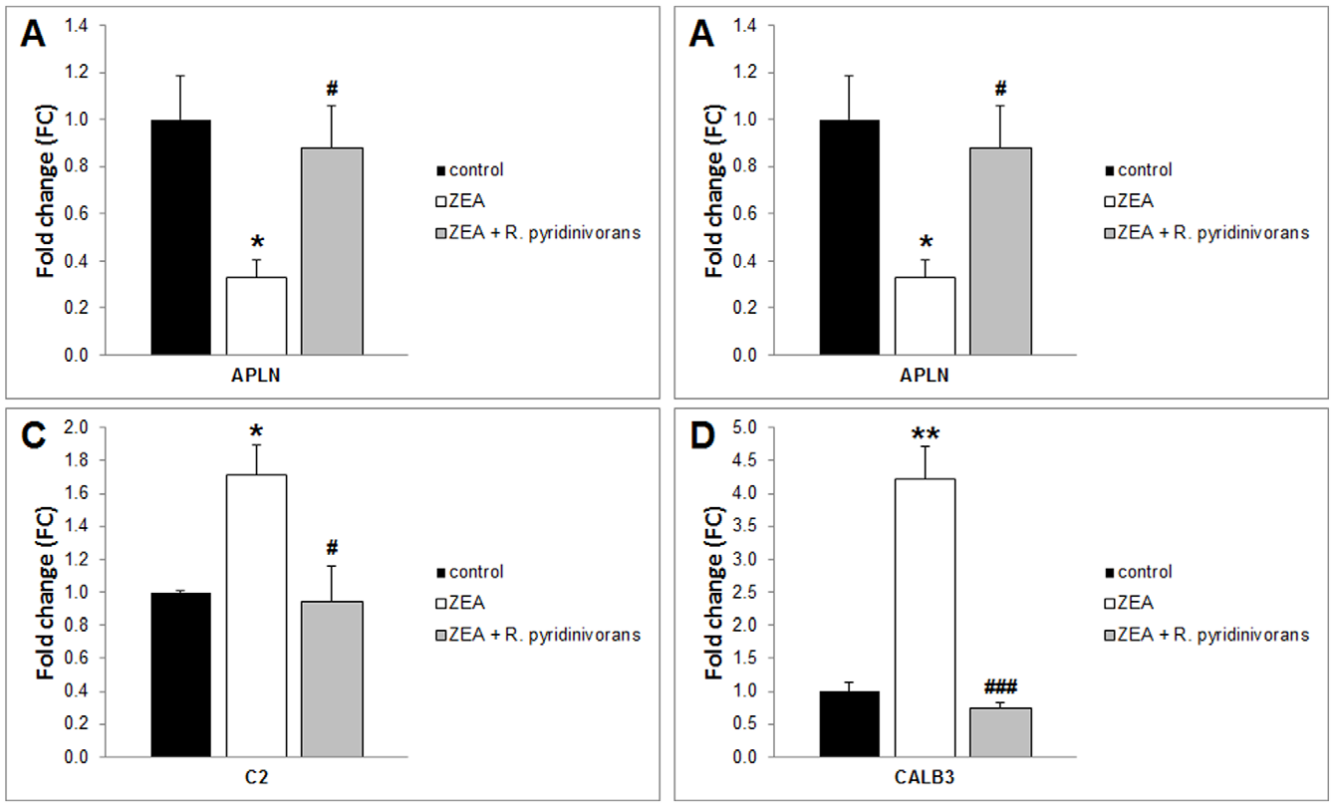
Gene expression of apelin, aquaporin 5, complement component 2, and calbindin 3 in *Experiment 2*. Gene expression of apelin (APLN) and aquaporin 5 (AQP5) decreased and complement component 2 (C2) and calbindin 3 (CALB3) increased significantly after 3-day ZEA treatment. There is no significant difference between control and ZEA + *R. pyridinivorans* K408 treatment in folding changes. (*) Significantly different from control (p<0.05). (**) Significantly different from control (p<0.01). (#) Significantly different from ZEA (p<005). (###) Significantly different from ZEA (p<0,001) (n = 6–7).

## Materials and Methods

### 2.1 Identification of strain K408

Genomic DNA was extracted from strain K408 using G-spinTM Genomic DNA Extraction Kit (Intron Biotechnology Inc., South Korea). For PCR-based 16 S rDNA multiplication universal primers 27F, and 1492R were used [23]. PCR products were purified with the Viogene DNA/RNA Extraction Kit (Viogene BioTek Corp., Taiwan). The almost complete 16 S rRNA gene sequence of strain K408 was determined by using BigDye Terminator v3.1 Cycle Sequencing Kit (Applied Biosystems, USA) in accordance with the instructions of the manufacturer. Sequencing products were separated on a Model 310 Genetic Analyzer (Applied Biosystems). Primers used for 16S rDNA sequencing reactions were 27F, 338F, 803F, 1492R [23]. The 16S rDNA sequence analysis were done by Blast tool, NCBI [24] and by the use of Le BiBi server [25].

**Figure 6:**
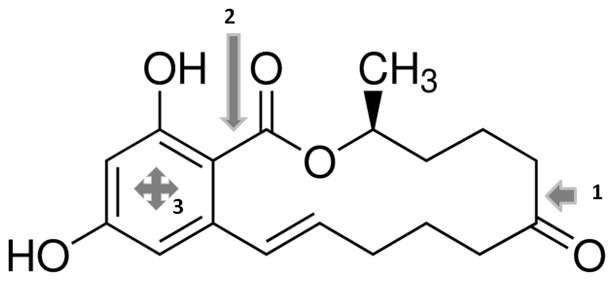
Putative mechanisms of the ZEA biodegradation by *Rhodococcus pyridinivorans* K408. The arising metabolites and the zearalenone degradation mechanism of *Rhodococcus pyridinivorans* K408 are unknown yet. However, previous studies suggest various ways how zearalenone could be degraded by microbes. *Trichosporon mycotoxinivorans* (**1**) and *Clonostachys rosea* IFO 7063 (**2**) are able to open the lactone ring resulting non-estrogenic metabolites which are 1-(3,5-dihydroxyphenyl)-10′-hydroxy-1-undecen-6′-one and (5S)-5-({2,4-dihydroxy-6-[(1E)-5-hydroxypent-1-en-1-yl]benzoyl}oxy) hexanoic acid, respectively. In addition, *Rhodococcus pyridinivorans* K408 have versatile metabolic pathways and the most of them are able to degrade aromatic compounds. It suggests that opening the aromatic ring is also a possible way to degrade zearalenone (**3**).

Almost complete (1355 bp) 16 S rDNA sequence of strain K408 was determined and submitted to GenBank under the accession number jx021450. According to the Blast analysis with all the type strains of rhodococci the highest similarity (99.85% ) was found with *Rhodococcus pyridinivorans* ^T^ DSM 44555. Phylogenetic analysis also confirmed the identification of the investigated strain as LeBibi alignment clearly placed strain K408 to *R. pyridinivorans* species. On the basis of genotypic data the zearalenone degrading bacterium was identified as *Rhodococcus pyridinivorans* K408.

### 2.2 Biodegradation of Zearalenone by *Rhodococcus pyridinivorans* K408

Cells of *Rhodococcus pyridinivorans* K408 strain were streaked on LB agar plates and incubated at 28°C for 72 hours. Single colonies were inoculated into 50 ml aliquots of, LB medium incubated at 28°C (170 rpm) for 48 hours. When the optical density of the cultures reached 0.6 (OD_550_ = 0.6), 15 ml of this culture was added into 135 ml sterile LB medium. ZEA (Fermentek, Israel), from acetone stock, was added to the medium to a final concentration of 5 μg/ml. As a control, 150 ml bacterium free LB medium with 5 μg/ml ZEA was used. The growth media and controls, in three parallels, were incubated in a shaking thermostat (170 rpm, maintained at 28°C, for 5 days). Every 24 hours, 1ml samples were taken and, centrifuged at 20.000×g, (4°C), for 20 min. Both supernatant and pellet were stored at −20°C until required. The remaining ZEA concentrations in samples of supernatant and pellet were analyzed by High Performance Liquid Chromatography (HPLC) and BLYES.

### 2.3 Quantifying the Zearalenone level by HPLC analyses

#### High-performance liquid chromatography (HPLC)

The sample was diluted with water and cleaned on a ZearalaTest immunaffinity column (VICAM). The ZEA was eluted with methanol from the column.

After dilution with methanol: water (1∶1) a 50 µl aliquot of the sample was injected to the HPLC-FLD system (liquid chromatography separation and fluorescence detection of toxins according to AOAC Official Method 985.18). The separation was carried out on a C18 column (Vydec Denali, 4.6×150 mm; 5 μm) using water: acetonitrile (45∶55) under isocratic conditions. The ZON was detected with extinction wavelength 274 nm and emission wavelength 440 nm.

### 2.4 Determination of the Estrogen effect modulated by ZEA in BLYES assay

The strain *Saccharomyces cerevisiae* BLYES, contains the human estrogen receptor on its chromosome and the luminescence gene cluster *lux* of *Photorhabdus luminescens* on two plasmids [20]. This enables the detection of EDC by a simple luminescence measurement.

*S. cerevisiae* strains BLYES were stored at −20°C and grown overnight at 30°C (200 rpm) to an OD_600_ of 0.1, in a modified minimal medium (YMM leu^-^, ura^-^) [26].

Supernatants of the biodegradation study were diluted five-fold, in order to evaluate the estrogen effect in the linear region of the dose-response curve of BLYES. Supernatants of ZEA biodegradation were examined in 1∶2 serial dilutions (sample concentration of 100–3.13%) for their remaining estrogenic effect. This was achieved by placing 20 µl of each of the biodegraded ZEA solution into 96-well micro-titer plates (Grenier Bio-one) and subsequently 200 µl of *S. cerevisiae* culture (BLYES) was added into each well. Bioluminescence was measured after 5 hours in a VictorX Multilabel Plate Reader (Perkin Elmer, Hungary). Negative controls included wells containing *S. cerevisiae* cultures (BLYES) and wells containing cultures and solvent (acetone) LB medium with 1 μg/ml ZEA was applied here as a positive control. For data analysis, luminescence was determined with the following modified formula (a):
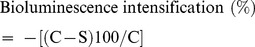
(a)where C is the arithmetic mean of the bioluminescence values of parallel controls, after incubation, and S represents the bioluminescence average value of parallel samples determined at the time of contact.

### 2.5 Experimantal Animals in ZEA administration

Prepubertal female Wistar rats from a colony bred at the Institute of Experimental Medicine (Budapest, Hungary) were used. Animals were kept under controlled conditions (3 animals per cage, at a temperature of 21±1°C, and humidity at 65%). The animals were illuminated for 12 hours per day (07:00–19:00 hours). Phytoestrogen free rat feed and untreated tap water were available *ad libitum*.

Rats were weaned, weighed and allocated to treatment groups at postnatal day 18 (PND 18). All experimental procedures were performed in accordance with the European Communities Council Directive of 24 November 1986 (86/609/EEC) and Hungarian Government directive 243/98. Experiments were approved by the Institutional Animal Care and Use Committee of the Institute of Experimental Medicine.

### 2.6 ZEA administration of female rats and the uterotrophic assay

An uterotrophic assay was performed using the validated Organisation for Economic Co-operation and Development 440 (OECD) protocol on prepubescent rat.

E2 and ZEA solutions were prepared as follows: 1 µg of 17β-estradiol, (E2, SIGMA) was dissolved in 50 µl 70% ethanol, and then mixed with 5 ml olive oil. 10 mg ZEA (Fermentek, Israel) was dissolved in 200 μl 96% ethanol then mixed with different volumes of olive oil. Drugs were administered with oral gavages (2 ml/kg body weight), daily starting at 09:00 hours. Treatment groups were as follows: E2 (0.04 mg/kg bw, positive control), ZEA (0.1-1-5-10 mg/kg bw) and the vehicle control (olive oil). Treatment started at PND 18 and lasted for 3 consecutive days (PND 18–20). At PND 21 rats were weighed and sacrificed by decapitation, trunk blood collected and the uteri dissected and weighed. One uterine horn was frozen immediately on dry ice, and stored at −70°C, whereas the other horn was fixed in 4% paraformaldehyde (0.1 M phosphate buffer) for histology.

Immediately after dissection, wet and blotted uterine weights were obtained. Wet weight includes the uterus and the luminal fluid contents. The blotted weight was measured after the luminal contents of the uterus have been removed (by pressing). Wet uterus weights were normalized to body weight.

### 2.7 Dose determination of Animal feeding experiments investigating the efficiency of ZEA biodegradation

The treatment groups were the following: LB broth (control); zearalenone-contaminated (500 ppm) LB broth; and zearalenone–contaminated (500 ppm) LB broth, which was treated with *R. pyridinivorans* K408. For the animal feeding experiments; *Rhodococcus pyridinivorans* K408 strain degraded ZEA samples were collected and centrifuged at 20.000×g (4°C) for 20 minutes. The supernatants were concentrated by a factor of 100 on an Edwards Micromodulyo lyophilisator and ZEA concentrations measured by HPLC as described above. ZEA content was also quantified by HPLC in the bacterium free ZEA sample.

For each individual animal, doses were calculated daily. ZEA intake was 5 mg/kg bw.

### 2.8 Blood ZEA concentration measurement by competitive fluorescent-microsphere based immunoassay (CFIA)

Plasma samples (250 µl) were incubated with β -glucuronidase (5000 U, Sigma Aldrich Ltd.) at 37°C for 24 h [27]. After glucuronidase treatment, the samples were frozen, at −20°C for 5 minutes then the samples were diluted 20 fold in phosphate buffered saline (PBS, pH 7.4) and loaded on a specific immunoaffinity Zearelenone column (IAC, ZearaStar COIAC 4000). The column was washed with 20 ml PBS/TrX.

#### Flow cytometric analysis

The measurment of ZEA concentration in the isolated fraction of the blood sample by the affinity column was performed using Fungi VI kit (Cat#503106, Soft Flow Hungary R& D Ltd., Pecs, Hungary), The *flow cytometric* measurements were performed on BD FACSArray^TM^ Bioanalyzer. Data analysis was carried out using FCAP 3.0 software.

### 2.9 The evaluation of the Estrogen-dependent gene expression at transcription level

For total RNA isolation uteri horns were homogenized in 500 μl TRI Reagent (Ambion). Total RNA was isolated using Total RNA Mini Kit (Geneaid) according to the manufacturer's instructions. To eliminate genomic DNA contamination DNase I treatment was introduced (1 U/reaction, reaction volume: 50 µl RNase-free DNase I, Fermentas). Sample quality control and the quantitative analysis were carried out by NanoDrop (Thermo Scientific). cDNA synthesis was performed with the High Capacity cDNA Reverse Transcription Kit (Life Technologies) according to the manufacturer's instructions.

The chosen primer sequences [22] used for the comparative C_T_ experiments were verified with the Primer Express 3.0 program. The sequences are as follows:

**Acidic ribosomal phosphoprotein P0** (ARBP; RefSeq: NM_022402):

Forward: CCT AGA GGG TGT CCG CAA TGT G

Reverse: CAG TGG GAA GGT GTA GTC AGT CTC

**Apelin** (APLN; RefSeq: NM_031612):

Forward: TCT CAC CCA CCA AGT TCC TCT AAT G

Reverse: GGT CTC CAA GGG CAG TCC AAG

**Aquaporin 5** (AQP5; RefSeq: NM_012779):

Forward: CCG CTT TGG AAT CAG GCA GAA TG

Reverse: TGG CAC TTG AGA TAC TGG GTT GG

**Complement C2** (C2; RefSeq: NM_172222):

Forward: CCA CCA ATC CCA TCC AGA AGA GG

Reverse: TGA GAG GCA TCA AGC AGC AGA TAG

**Calbindin-3** (CALB3; RefSeq: NM_012521):

Forward: TAG GAT TCA ATC AGT AGG TGG TGT CG

Reverse: GAT AAG AAC GGT GAT GGA GAA GTT AGC

The primers (Invitrogen) were used in real-time PCR reaction with Fast EvaGreen qPCR Master Mix (Biotium, USA, CA) on an ABI StepOnePlus instrument. The gene expression was analyzed using the ABI StepOne 2.1 program. The amplicons were tested by Melt Curve Analysis on an ABI StepOnePlus instrument. Experiments were normalized to ARBP expression.

### 2.10 Statistical analysis

Data are presented as mean ± S.E.M. Statistical significance was calculated with one-way ANOVA and after that we checked the differences with the Dunnet's post hoc test. For multiple comparisons were used Statistica 8.0 software. For the correlation- and multiple regression analyses were used the MedCalc® 10 software. Differences with a P-value <0.05 were considered significant.

## Results

### 3.1 Degradation experiments to analyze ZEA biodegradation efficiency by HPLC and BLYES

Analyses of the pellet by HPLC detected lower than 1% residual ZEA content of the original concentration (5 μg/ml), thus ZEA elimination from the matrix was attributed to metabolic activity. At the end of the 120 hours (5 days) incubation period 0.53±µg/ml ZEA concentration was measured from the culture supernatant by HPLC. This represents 87.21% (± 2.83) degradation efficiency (data not show).

The result of the BLYES based estrogenic biotest showed good correlation with the analytical ZEA concentration measurement at the end of the 5^th^ day (Fig. 1). The analytical method showed 87.21% ZEA degradation in the supernatant of K408, while BLYES showed a reduction of 81.75% in estrogen effects.

The time course of the ZEA degradation shows that after 24 hours, *R. pyridinivorans* K408 eliminated 44.64% of the estrogenic effects of ZEA. Then the concentration remained stable for 72 hours, after which the BLYES tester strain indicated 56% decrease in biological activity. The degradation developed by the fifth day of the experiment reaching 81.75%.

### 3.2 Animal studies for detecting the effect of different ZEA dose administration (*Experiment 1)*

In *Experiment 1* the effects of zearalenone have been examined on body weight, uterus weight and on the expression of estrogen sensitive genes. In addition, plasma ZEA concentration was measured by CFIA.

#### 3.2.1 Effect of ZEA on body weight

3 days administration of ZEA did not result in any significant change in body weight: The average body weight gain during the experiment was 11.08±0.58 g and was not different in the different groups.

#### 3.2.2 Blood ZEA concentration measurement by CFIA

As shown on Figure 2, there was a dose dependent increase in plasma ZEA concentration following oral administration of the mycotoxin. The lowest dose (0.1 mg/kg) did not result in an increase of ZEA level in the plasma following 3 days exposure compared to vehicle treated control prepubertal female rats. The 1 mg/kg bw dose caused significantly elevated blood ZEA levels (39.69±10.20 ng/ml ANOVA, F.:20.49, p = 0.006). The 5 mg/kg bw administration caused yet higher ZEA levels in blood plasma (ANOVA: F = 8.59, p = 0.03). The highest concentration of ZEA (10 mg/kg bw) did not significantly increase further the blood plasma levels of ZEA (ANOVA: F = 2.51, p = 0.16) compared to the 5 mg/kg ZEA group. Blood plasma levels of ZEA showed a strong positive correlation (R  = 0.919 p<0.01) with uterus weight in a dose dependent manner (not shown).

#### 3.2.3 Uterotrophic assay

Treatments of ZEA in 0.1 and 1 mg/kg bw doses had no significant effect on uterine weight. In contrast, the 5 and 10 mg/kg bw ZEA and 0.04 mg/kg bw E2 administration resulted in increased relative uterine weight. Positive control treatment (E2; 0.04 mg/kg bw) and highest dose ZEA (10 mg/kg bw) resulted in approximately 3.8-fold increase of uterine weight. Administration of ZEA in 5 mg/kg bw doses resulted in a 2.4-fold increase (Fig. 3A).

#### 3.2.4 Estrogen-dependent gene expression

Four validated genes were chosen for use in this study, with regards to Heneweer et al. [22]. These genes were apelin (APLN), aquaporin 5 (AQP5), complement component 2 (C2) and calbindin-3 (CALB3). Initially the suitability of this method was verified by using ZEA at 10 mg/kg bw and E2 at 0.04 mg/kg bw (*Experiment 1*). For this purpose we presented a quantitative real-time PCR measurement with the samples using the same oligonucleotide primers used in Heneweer's study, as described. Gene expression levels of APLN and AQP5 are decreased following ZEA and E2 treatment (Fig 4 A, B). Expression of C2 and CALB3 genes are increased following ZEA and E2 treatment (Fig. 4C, 4D).

### 3.3 Evaluating of ZEA biodegradation (*Experiment 2*)

#### 3.3.1 Effect of ZEA on body weight

The *Experiment 2* demonstrated the effect of microbially decontaminated ZEA, and its metabolites, to female prepubertal rats. ZEA containing LB medium with or without *R. pyridinivorans* K408 had no significant effect on body weight gain.

#### 3.3.2 Evaluating of ZEA biodegradation with uterotrophic assay

In *Experiment 2.,* following the experience gained in *Experiment 1,* uterine weight as a morphological marker was monitored. A significant increase in uterine weight (2.6-fold as compared to the control group) was noted following treatment with non-incubated zearalenone solution (ZEA). The other two experimental groups (control and ZEA + *R. pyridinivorans*) yielded similar results. Comparing the results of the ZEA group with the ZEA + *R. pyridinivorans group*, it can be established that incubation with the microbe decreased the uterotrophic effect by 2.6-fold (Fig. 3B).

#### 3.3.3 Evaluating of ZEA biodegradation with estrogen-dependent gene expression

Results confirmed that incubation with *R. pyridinivorans* K408 decreased estrogen-dependent effects of zearalenone-contaminated LB media. Treatment with ZEA resulted in similar alterations in gene expression levels as demonstrated in *Experiment 1.* Expression levels of APLN and AQP5 are decreased whereas expression levels of C2 and CALB3 are increased significantly (Fig. 5). However there are no significant differences in folding change between control and ZEA + *R. pyridinivorans* treatment.

## Discussion

This study has demonstrated that *R. pyridinivorans* K408 efficiently degrade ZEA without producing active estrogenic metabolites.

There are different physical, chemical and biological strategies for Zearalenone (ZEA) decontamination, such as adsorbents [28] pulsed light technology [29], ozone gas oxidation [30], plantibodies [31] and cropping genetically modified maize that produce ZEA-degrading enzymes [32]. Biodegradation seems to be the most promising approach, with regards to ZEA decontamination. There are several reports on ZEA biodegradation by microorganisms including bacteria and fungi, however vast majority of these transformations cannot be regarded as detoxification since the estrogenic activities of these metabolites were not elucidated or remained similar to that of ZEA [33]. Among those microbial examples with known effective biodegradation properties, where detailed measurements of toxicological and estrogenic effects of the metabolites were performed, is the case of fungus *Trichosporon mycotoxinivorans*. In this case, the loss of estrogenic activity of ZEA and its derivatives was tested by simply using estrogen bioindicator yeast strain YZRM7. Animal models were not used [34]. Similarly, the estrogenic effects of ZEA degradation using *Clonostachys rosea,* was not investigated living animal models [35]. A further example, using the eubacterial *Pseudomonas putida* ZEA-1 strain, ZEA degradation ability was investigated only by use of the *Artemia salina* based toxicity bioassay [16]. The use of an animal model for testing the EDC effects of the arising metabolites increases the reliability of monitoring the mycotoxin degradation.

This study utilized several analytical techniques and different bioassays to reveal the endocrine modifying effects of ZEA degradation products. First, the efficiency of ZEA-degradation was measured by analytical methods. The ZEA-concentration of supernatant, which arose during biodegradation, was measured by HPLC-FLD which demonstrated efficient degradation. However, the use of HPLC alone could not provide information about bioactive properties of degraded mycotoxin. Consequently, in-vitro methods are needed.

ZEA (and other estrogenic compounds) stimulates the proliferation of MCF-7 human adenocarcinoma cells [7] where estrogenic activity is proportional to neoplastic cell number. Detection could be significantly simplified with transfected cell lines which contain estrogen receptor genes and a reporter gene construct. The disadvantage of this method is the usage of mammalian cells which could be more complicated than bacteria and yeasts. This drawback can be avoided through the use of the luciferase assay. In this study BLYES was used which is suitable for high-throughput screening of estrogenic molecules and ZEA-degrading microbes. The animals metabolize ZEA into two major metabolites, α-zearalenol (α-ZOL) and β-zearalenol (β-ZOL), which are also ligands of ER [6]. The metabolite α-ZOL shows a higher estrogenic activity than ZEA, by contrast β-ZOL was less effective in ER binding assay [36]. Our results showed that incubation the zearalenon contaminated bacterial media (LB) with *Rhodococcus pyridinivorans* K408 did not increased the bioluminescence of yeast cells, the indicators of estrogenic activity. It seems that *R. pyridinivorans* K408 metabolizes ZEA without the production of estrogenic compounds, such as α- and β-ZOL, These results were confirmed with a bioassay using prepubertal rat uterotrophic assay. The animal experiments demonstrated that change in uterine weight is unequivocal evidence of an estrogenic effect. The circulating level of ZEA, and its metabolites, in the blood showed a positive correlation with the uterine weight. The enterohepatic ZEA circulation maintains a steady-state zearalenone concentration and increases the toxic effect of the ZEA and its derivates via extend their biological half-life [37]–[38]. A previous study examined the changes in expression of estrogen-responsive genes (APLN, AQP5, C2 and CALB3) via mRNA level changes using qRT-PCR [22]. In this study, gene expression levels of APLN and AQP5 are decreased following treatment with ZEA and E2 while expression of C2 and CALB3 genes show an increase following ZEA and E2 treatment in Experiment 1. The uterotrophic bioassay demonstrated no significant change in expression of the investigated genes in the uteri of the control and ZEA + *R. pyridinivorans* K408 treated animals in Experiment 2. The endocrine disruptor effect of ZEA and its derivatives are eliminated during incubation with *R. pyridinivorans* K408. There are few microorganisms able to metabolize ZEA and eliminate efficiently their endocrine modifying effect. The details of the degradation mechanisms were presented the *Clonostachys rosea and Trichosporon mycotoxinivorans* species alone.The plant pathogen *Clonostachys rosea (*previously known as *Gliocladium roseum*) NRRL 1859 strain was capable of metabolizing zearalenone in 80–90% yields. The product was a 1:1 mixture of 1-(3,5-dihydroxyphenyl)-10′-hydroxy-1-undecen-6′-one and 1-(3,5-dihydroxyphenyl)-6′-hydroxy-1-undecen-10′-one which were determined via spectroscopy analysis. These metabolites showed far less estrogenic effects then ZEA [39] Kakeya and coworkers isolated another strain, *Clonostachys rosea* IFO 7063, which is able to degrade zearalenone without arising estrogenic metabolites. They could purify the lactonohydrolase enzyme (*zhd101*) which is responsible for the degradation mechanism [35]. The structure of the arising metabolite (1-(3,5-dihydroxyphenyl)-10′-hydroxy-1-undecen-6′-one) was also determined by 2D NMR spectroscopy. This compound did not show estrogenic activity in the human breast cencer MCF-7 cell proliferation assay [40]. The yeast strain *Trichosporon mycotoxinivorans* is also capable to efficiently degrade zearalenone to non-estrogenic compounds [41]. The metabolite was identified as (5S)-5-({2,4-dihydroxy-6-[(1E)-5-hydroxypent-1-en-1-yl]benzoyl}oxy) hexanoic acid via NMR spectroscopy. This metabolite did not interact with the human estrogen receptor in an *in vitro* competitive binding assay [34]. The identified cleavage positions of the *Clonostachys rosea* and *Trichosporon mycotoxinivorans* strain?s enzymes on the ZEA molecule were summarised in Figure 6. These data suggest that one putative mechanism of the zaearalenone degradation by *Rhodococcus pyridinivorans K408* could be the opening of the lactone ring in the ZEA molecule, losing of their estrogenic activity. On the other hand it is also possible that the microbe could open up the aromatic ring as the strains of *Rhodococcus pyridinivorans* have versatile metabolic pathways and the most of them are able to degrade aromatic compounds like pyridine, styrene and BTEX (benzene, toluene, ethylbenzene, and xylenes) chemicals as well [42], [43], [44], [45], [46] (Fig. 6).

The second achievement in this study is the application of a novel microbe which is not only able to degrade ZEA effectively, but also bioactive estrogenic metabolites did not arise during biodegradation. Consequently *Rhodococcus pyridinivorans* K408 can be used to decontaminate raw materials, and foodstuffs, by reducing the ZEA concentration. After elucidating the metabolic pathway of ZEA and isolating the enzymes which participate in its degradation, an additive could feasibly be developed and thus reducing the harmful effects on livestock. It is not only feed can be decontaminated using the microbe but also distillers dried grains with soluble (DDGS) occurred by bioethanol production from maize [47].
